# Metabolic and Homeostatic Changes in Seizures and Acquired Epilepsy—Mitochondria, Calcium Dynamics and Reactive Oxygen Species

**DOI:** 10.3390/ijms18091935

**Published:** 2017-09-08

**Authors:** Stjepana Kovac, Albena T. Dinkova Kostova, Alexander M. Herrmann, Nico Melzer, Sven G. Meuth, Ali Gorji

**Affiliations:** 1 Department of Neurology, University of Münster, 48149 Münster, Germany; alexander.herrmann@ukmuenster.de (A.M.H.); nico.melzer@ukmuenster.de (N.M.); sven.meuth@ukmuenster.de (S.G.M.); gorjial@uni-muenster.de (A.G.); 2 Division of Cancer Research, School of Medicine, Jacqui Wood Cancer Centre, Ninewells Hospital and Medical School, University of Dundee, Dundee DD1 9SY, UK; A.DinkovaKostova@dundee.ac.uk; 3 Departments of Medicine and Pharmacology and Molecular Sciences, Johns Hopkins University School of Medicine, Baltimore, MD 21205, USA; 4 Shefa Neuroscience Research Center, Khatam Alanbia Hospital, Tehran 1996836111, Iran; 5 Department of Neuroscience, Mashhad University of Medical Sciences, Mashhad 9177948564, Iran; 6 Department of Neurosurgery, University of Münster, 48149 Münster, Germany; 7 Epilepsy Research Center, University of Münster, 48149 Münster, Germany

**Keywords:** epilepsy, reactive oxygen species, mitochondria, Nrf2, calcium, cell death

## Abstract

Acquired epilepsies can arise as a consequence of brain injury and result in unprovoked seizures that emerge after a latent period of epileptogenesis. These epilepsies pose a major challenge to clinicians as they are present in the majority of patients seen in a common outpatient epilepsy clinic and are prone to pharmacoresistance, highlighting an unmet need for new treatment strategies. Metabolic and homeostatic changes are closely linked to seizures and epilepsy, although, surprisingly, no potential treatment targets to date have been translated into clinical practice. We summarize here the current knowledge about metabolic and homeostatic changes in seizures and acquired epilepsy, maintaining a particular focus on mitochondria, calcium dynamics, reactive oxygen species and key regulators of cellular metabolism such as the Nrf2 pathway. Finally, we highlight research gaps that will need to be addressed in the future which may help to translate these findings into clinical practice.

## 1. Introduction

Epilepsy, a devastating disease, affects over 50 million people worldwide [1] and is defined by the occurrence of unprovoked seizures. Most of these epilepsies are acquired as a consequence of a brain injury and are followed by a latent period of epileptogenesis [2]. Once unprovoked seizures occur, the patient is diagnosed with epilepsy and anticonvulsive treatment is initiated. Many patients become seizure free with antiepileptic drugs, although approximately one third of patients develop pharmacoresistant epilepsy [3], highlighting the unmet need for new treatment strategies. Current anticonvulsants mainly act on neuronal voltage gated ion channels, whereas downstream signaling cascades and non-neuronal cells are not targeted directly. However, the latter may be instrumental in mediating pharmacoresistance and also epilepsy comorbidities. It is likely that downstream signaling cascades such as metabolic and homeostatic cellular mechanisms contribute to epileptogenesis, fully established epilepsy and pharmacoresistant epilepsy, although the precise mechanisms remain unclear.

There are clinical hints pointing to a strong involvement of mitochondria and bioenergetics in epileptogenesis, seizures and epilepsy. For example, patients with mitochondrial mutations often present with epilepsy as a phenotypic manifestation of the disease [4], highlighting the involvement of mitochondrial dysfunction in epileptogenesis. In addition, seizure activity and epilepsy have been linked to energy failure, which has been hypothesized to lead to neuronal injury responsible for the clinical sequelae associated with epilepsy patients. The brain, which makes up only 2% of the total bodyweight, contributes up to 20% to the resting whole body metabolism. This large metabolic turnover is mainly due to synaptic transmission [5,6] where vesicle cycling consumes the majority of presynaptic adenosine triphosphate (ATP) to mediate neuronal function [7]. Epileptiform activity also induces large ionic conductances and depletes vesicular stores. Restoration of these changes, i.e., restoration of cellular homeostasis, is an energy demanding process [8]. Thus, it is not surprising that ATP demand and production during seizures, epilepsy and particularly prolonged seizures, such as those seen in status epilepticus, the maximum expression of epilepsy, is critical. It should be noted though that mitochondria are not the only source of dysfunction in epileptic seizures, and there are also other targets of homeostatic imbalances that occur during seizure activity. The most prominent homeostatic changes during seizure activity include the accumulation of intracellular calcium and the increased production of reactive oxygen species (ROS). Neuronal compromise during seizure activity is dependent on intracellular Ca^2+^ entry [9]. There is accumulating evidence that *N*-methyl-d-aspartate (NMDA) receptors play a pivotal role in intracellular Ca^2+^ accumulation during seizure activity. This is supported by robust evidence showing that blocking of NMDA receptor activity abolishes cell death both in vitro and in vivo [10,11,12]. NMDA receptor opening has also been shown to promote ROS production via nicotinamide adenine dinucleotide phosphate (NADPH) oxidase, an enzyme which has recently been drawn into the spotlight of seizure induced neuronal damage and neuronal autophagy [13,14]. Excess ROS and Ca^2+^ are potent triggers of the mitochondrial permeability transition pore opening, which is a key event and point of no return leading to mitochondrial swelling and cytochrome c release from mitochondria, subsequently triggering the cell death cascade [15,16,17].

In this review, we summarize current knowledge about metabolic and homeostatic changes in seizures and acquired epilepsy with a particular focus on mitochondria, Ca^2+^ dynamics, ROS and key regulators of cellular metabolism such as the nuclear factor erythroid 2–related factor 2 (Nrf2)pathway. It is not in the scope of this review to cover the extensive literature on genetic syndromes with mutations in genes coding for mitochondrial proteins or key enzymes of metabolism, which has been the subject of a number of other extensive reviews (e.g., [4,18]). Instead, we wish to focus on patients suffering from acquired epilepsies (e.g., such as epilepsy due to hippocampal sclerosis) who are frequently encountered in clinical practice and pose a challenge to practitioners given that 30% present with pharmacoresistant seizures [3,19]. Finally, we highlight research gaps that will need to be addressed in the future which may help to translate findings into clinical practice.

References for this review were identified through searches of PubMed with combinations of the terms from keywords from the subsection titles (e.g., mitochondria) and “epilepsy” or “seizures” from 1950 until July 2017. In addition, manuscripts were also identified through searches of the authors’ own files and from reference lists of the articles pointed out by the PubMed searches. Due to space restrictions, the final reference list was compiled from a selection of the articles identified which were prioritized according to their originality and their ability to fit into the narrative style of the current review.

## 2. Mitochondria and Epilepsy—Adenosine Triphosphate (ATP), Ca^2+^ and Cell Death

Mitochondria, initially coined bioblasts, were first described by Richard Altmann in 1890 and were recognized to function as elementary organisms within the cell [20]. A further advance in their functional characterization came through the introduction of new methods to study electron transport and metabolic states of the respiratory chain [21,22,23,24]. Peter Mitchell introduced the chemiosmotic hypothesis of oxidative phosphorylation in 1961, linking energy metabolism to hydrogen transport across the mitochondrial membrane [25]. Oxidative phosphorylation is the main source of ATP generation in neurons since they lack powerful enzymes for glycolytic ATP production such as those that are available in astrocytes [26].

The mitochondria are organelles that are amongst others essential in three different homeostatic mechanisms. Firstly, their most prominent and obvious function is ATP production. Secondly, they are involved in Ca^2+^ homeostasis through buffering of intracellular Ca^2+^, and thus have been referred to as the “hub of Ca^2+^ signaling” [27]. Lastly, mitochondria are instrumental in apoptotic cell death amongst others through the release of intramitochondrial cytochrome c [28]. Links to seizures and epilepsy have been established for all three mechanisms.

### 2.1. ATP during Seizures and Epilepsy

Early pioneering experimental studies showed that glucose, ATP and other energetic substrates decrease during seizure activity, particularly if this is prolonged [29,30,31]. This has been confirmed on a cellular level, where ATP decrease during seizure activity has been shown in neurons [12]. Mitochondrial dysfunction and seizures have been closely linked to each other not only in epilepsies, i.e., mitochondrial epilepsies, but are also increasingly recognized as a target in acquired epilepsy [18]. Anaplerosis, a strategy that aims to restore mitochondrial function through providing tricarboxylic acid cycle substrates, has been recently advocated for use in acquired epilepsy [32]. The ketogenic diet, a high fat diet used for the treatment of epilepsies, has been shown amongst other effects to upregulate the neuronal expression of genes involved in the tricarboxylic acid cycle, oxidative phosphorylation and glycolysis, along with improving mitochondria complex activities and boosting mitochondrial biogenesis [33,34,35,36]. The ketogenic diet is particularly the treatment of choice in patients suffering from seizures due to glucose transporter 1 (GLUT-1) deficiency syndrome and pyruvate dehydrogenase complex deficiency, given that it circumvents the metabolic deficiencies in these syndromes [37]. However, the ketogenic diet has also been found to be an effective treatment in syndromes which are not directly linked to mutations in the metabolic pathway, such as Dravet syndrome or Doose syndrome [37,38] and has been advocated for the treatment of epilepsies due to mitochonridal diseases [39]. The exact mechanisms of the seizure suppressive effect of the ketogenic remain elusive. Beside its anaplerotic properties and its effect on mitochondria, direct anti-seizure effects of ketone bodies, neurotransmitter and ion channel regulation as well as regulation of ROS amongst others have been shown to play a role [36].

### 2.2. Mitochondria and Ca^2+^ Buffering in Seizures and Epilepsy

Besides ATP production, the most important function of mitochondria is the buffering of excess Ca^2+^. Excess Ca^2+^ entry into neurons has been observed in rats that underwent status epilepticus induced by bicuculline and l-allylglycine [9]. In these studies, mitochondrial Ca^2+^ overload has been shown to lead to cell death and was particularly pronounced in CA1 and CA3 regions, which are areas of the central nervous system (CNS) that are very susceptible to seizure induced cell death [9,40]. It is interesting that these very early studies already identified mitochondria as the main site of Ca^2+^ accumulation during prolonged seizures.

**Figure 1:**
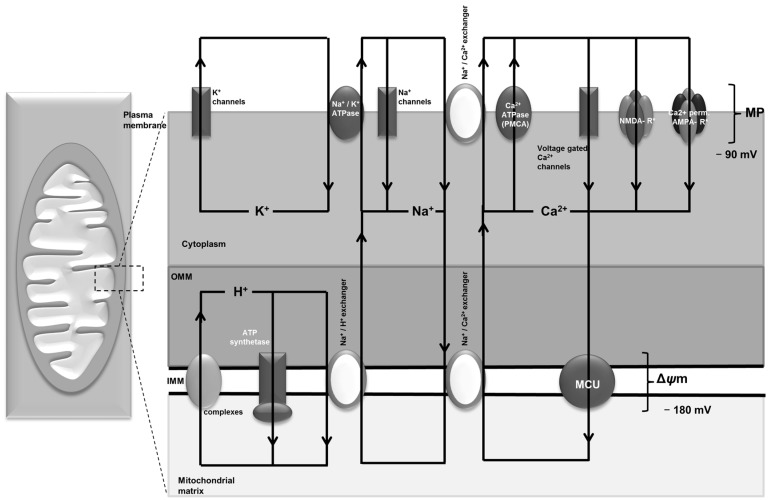
Ion circuits across mitochondrial and plasma membranes of a neuron. The figure shows a schematic drawing of ion circuits across mitochondrial and plasma membranes of a neuron. The respiratory chain is the driving force of these circuits either directly or indirectly by providing adenosine triphosphate (ATP) for all ATP-dependent processes [44]. MCU: mitochondrial Ca^2+^ uniporter; Δψm: mitochondrial membrane potential; MP: membrane potential; NMDA-R: *N*-methyl-d-aspartate receptor, AMPA-R: α-amino-3-hydroxy-5-methyl-4-isoxazolepropionic acid receptor; OMM: outer mitochondrial membrane; IMM: inner mitochondrial membrane; PMCA: plasma membrane Ca^2+^ ATPase.

Different pathways for mitochondrial Ca^2+^ buffering have been described in the literature. The mitochondrial uniporter (MCU), whose structure has been unraveled only recently [41], is the main site of Ca^2+^ entry into the mitochondria and is thus instrumental in Ca^2+^ buffering during excess Ca^2+^ overload. The uptake and extrusion of Ca^2+^ in mitochondria is coupled to H^+^ and Na^+^ cycling that is maintained by the electron transport chain, which creates a potential gradient across the mitochondrial membrane [27,42] (Figure 1). Within the mitochondrial membrane, Ca^2+^ precipitates to insoluble Ca^2+^ phosphate complexes, a process which is dependent on the availability of phosphate. This in turn is also dependent on the proton gradient and thus more phosphate enters the mitochondria if the gradient is higher, i.e., when the mitochondria are hyperpolarized. Ca^2+^ homeostasis through the MCU has been shown to play a role during seizure activity. Inhibition of the MCU significantly attenuated neuronal death after pilocarpine-induced status epilepticus and reduced levels of intracellular ROS [43].

**Figure 2:**
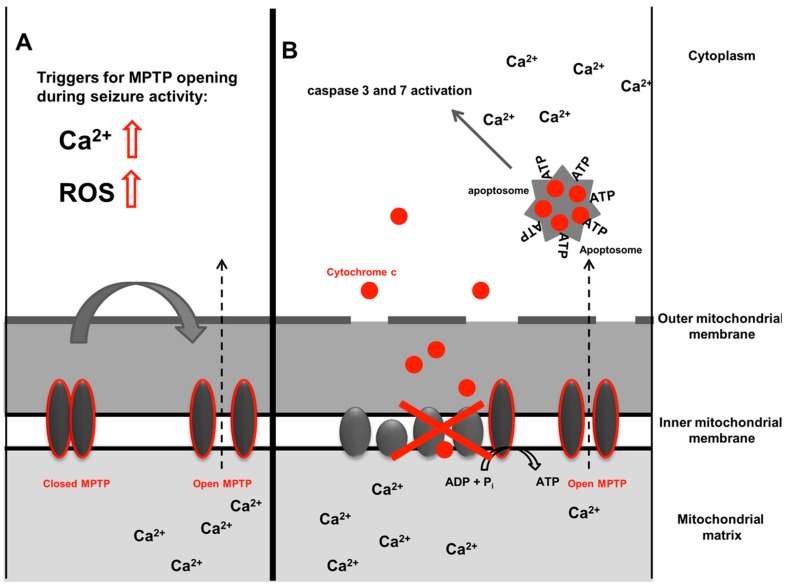
Mitochondrial permeability transition pore (MPTP) opening. Simplified model of the mitochondrial permeability transition pore. Permanent opening of the permeability transition pore leads to mitochondrial outer membrane permeabilization (MOMP). ADP: adenosine diphosphate; Pi: phosphate group; ROS: reactive oxygen species. Reduced function of the respiratory chain, as indicated with the red arrow (in B) subsequently leads to mitochondrial disintegration and release of cytochrome c.

Mitochondrial permeability transition pore (MPTP) opening is another route for Ca^2+^ trafficking across the mitochondrial membrane (Figure 2). The MPTP is a protein complex in the inner mitochondrial membrane which has long been a mystery, but was recently found to be formed of two ATP synthase (Complex V) dimers [45,46]. The importance of MPTP opening was soon recognized in the triggering of apoptotic cell death through an increase in permeability of Ca^2+^ ions [16,46]. Our group has shown that seizure-induced cell death is partly mediated via MPTP opening as treatment with cyclosporine A, a potent inhibitor of MPTP opening, was able to reduce mitochondrial depolarization, the initial event leading to cell death during seizure activity [12]. More recently, an interesting link between the MPTP and epilepsy treatment was made. Ketone bodies, which are the main effectors of anti-seizure effects in the ketogenic diet and are a very effective anti-seizure treatment, have been shown to mediate anti-seizure effects via MPTP modulation [47]. In this study, mitochondria isolated from hippocampi of *Kcna1*-null mice with a genetic susceptibility to seizures had a higher threshold for Ca^2+^ induced mitochondrial permeability transition if they were pre-treated with ketone bodies.

### 2.3. Cell Death, Mitochondria and Epilepsy

Early pioneering experiments by Meldrum and colleagues in baboons showed that brain seizure activity can cause neuronal damage even in the absence of a systemic convulsion, showing that brain damage is not only a secondary phenomenon which occurs due to excessive muscle activation and subsequent lactate acidosis [48]. That seizure activity itself can cause neuronal injury is now widely accepted and it is appreciated that even non-convulsive status epilepticus can be a life threatening emergency that is often encountered in certain clinical settings such as the intensive care unit [49]. Mitochondria have now been put into the spotlight of seizure induced cell death. Continuous seizure activity leads to ATP depletion and subsequent cell death [12,50]. Excess Ca^2+^ accumulation results in mitochondrial swelling, permeability transition pore opening, activation of mitochondrial proteins that trigger cell death (Bcl-2 family proteins Bax and Bok proteins [51]), release of cytochrome c, activation of caspases which lead to mitochondrial permeability transition pore opening and loss of mitochondrial outer membrane integrity [16,52,53,54,55,56]. Abnormal mitochondrial distribution, altered mitochondrial motility, decreased mitochondrial membrane potential, and diminished mitochondrial respiration was observed in fibroblasts derived from two lethal encephalopathic patients with a loss of function TRAK1 (trafficking of kinesin proteins 1) variant that was also shown to be associated with seizures [57].

## 3. Excess Intracellular Ca^2+^ during Seizure Activity—The Endoplasmic Reticulum

Excess intracellular Ca^2+^ accumulation, which is one of the leading causes for seizure induced sequelae, is an event that is not only governed by the interplay of Ca^2+^ entry through the plasma membrane, but also by intracellular Ca^2+^ stores. An intracellular site of Ca^2+^ accumulation resides, as mentioned above, within the mitochondria. However, the endoplasmic reticulum (ER) has a larger capability to store intracellular Ca^2+^ and thus is pivotal in Ca^2+^ homeostasis.

The ER, which was first identified as the site for intracellular Ca^2+^ release for muscle contraction [58,59], was soon accepted to regulate Ca^2+^ homeostasis in cells other than muscle cells [60]. Inositol triphosphate (IP3) mediated mechanisms of Ca^2+^ release have been shown to play a role in many neurological diseases [61].

With regards to epilepsy, surprisingly very few studies have examined the role of ER Ca^2+^ stores. Preincubation of neuronal hippocampal cultures with thapsigargin, a drug that depletes intracellular ER Ca^2+^ stores, resulted in a decrease in the amount of neuronal excitation produced by bicuculline [62]. Ictal discharges induced by pilocarpine or (RS)-3,5-dihydroxyphenylglycine in hippocampal slices were dependent on internal Ca^2+^ stores as they were blocked by thapsigargin or dantrolene, which both affect ER-Ca^2+^ stores [63]. A recent study showed that genetic silencing of nitric oxide-induced activation of the ryanodine receptor, a receptor triggering Ca^2+^ release from the ER, provides protection against cell death produced by kainate-induced status epilepticus [64]. This study highlights the importance of ER Ca^2+^ stores in mediating seizure-induced cell death.

In contrast to Ca^2+^ release from intracellular stores, extracellular Ca^2+^ entry has been identified as the most important route of Ca^2+^ entry during seizure activity, which is supported by a plethora of studies [11,12].

## 4. Ca^2+^ Channels and Transporters in the Plasma Cell Membrane and Their Role in Epilepsy—NMDA Receptor, AMPA Receptor, VGCC and PMCA

The NMDA receptor (NMDA-R) forms a heterotetramer comprising two GluN1 and two GluN2 subunits and is mainly permeable by Na^+^ and Ca^2+^ ions. Depolarization of the cell opens the channel by dislodging Mg^2+^ and Zn^2+^ ions from the pore [65]. The NMDA receptor has been shown to be instrumental in seizure-induced neuronal death both in vitro [11,12] and in vivo [66,67]. The importance of NMDA receptors in epilepsy is underpinned by the fact that NMDA receptor hyperactivation and upregulation of NMDA regulatory subunits is found in focal cortical dysplasia, a highly epileptogenic lesion [68,69]. Ca^2+^ ions entering the cell upon NMDA receptor stimulation have been shown to be responsible for subsequent cell death promoted by NMDA receptor activation, since omission of Ca^2+^ ions from the extracellular solution mitigated the harmful NMDA receptor mediated effects [11].

However, clinical evidence points to a more complicated role of NMDA receptors in epilepsy. Antibodies against the extracellular N-terminal domain of the GluN1 subunit have been identified to be responsible for NMDA receptor encephalitis [70,71], an antibody mediated disease which commonly presents with seizures. It remains an unresolved paradox though, why reduction of surface NMDA receptors as seen in anti-NMDA-R encephalitis leads to seizures. In this context, it is interesting that, despite a dramatic clinical presentation of anti-NMDA-receptor encephalitis, including status epilepticus, only a paucity of inflammatory markers and neuronal cell death have been observed, which has repeatedly been confirmed in pathological brain specimens of affected patients [72,73,74].

Interestingly, mutations in subunits of the NMDA receptor, e.g., GRIN1 and GRIN2B, have recently been identified as a cause of epileptic encephalopathy presenting with seizures [75,76]. In addition, the *NR1*^neo/neo^ mouse model of NMDA receptor hypofunction showed a dramatic sensitivity to kainate induced seizures [77], highlighting the proconvulsant effect of NMDA receptor dysfunction. It has been suggested that NMDA hypofunction may play a role in neurotoxicity during seizure events [78].

Besides NMDA receptors, Ca^2+^ permeable AMPA receptors have been shown to play a role in status epilepticus [79]. How substantial their contribution is to Ca^2+^ mediated injury in seizures remains to be determined.

A review of the role of voltage gated Ca^2+^ channels (VGCC) channels is beyond the scope of this review. It should just be mentioned though that T-type and P/Q-type channels contribute to epileptogenesis, modulation of network activity, and genetic seizure susceptibility [80]. These channels have been linked to genetic forms of epilepsy and idiopathic epilepsy such as childhood absence epilepsy. Rodent genetic models of absence epilepsy have revealed that Ca_V_3.1 and Ca_V_3.2 T-type channel isoforms of VGCC are essential in the pathogenesis of absence epilepsy [81]. In addition, subsequently these channels have also been shown to play a role in acquired epilepsy [82,83].

While NMDA receptors, Ca^2+^ permeable AMPA receptors and voltage gated Ca^2+^ channels mediate the influx of Ca^2+^ into the cell, the plasma membrane ATPase (PMCA) together with the sodium Ca^2+^ exchanger (NCX) removes Ca^2+^ from the cell against its concentration gradient (Figure 1). A decrease of Ca^2+^-ATPase activities was found in pentylentetrazole treated rats [84]. The expression of the Ca^2+^ extrusion proteins (PMCA and NCX) has been studied in a rat model of kainate-induced status epilepticus [85]. This study found differences of PMCA and NCX isoform expression in neurons and astrocytes in hippocampal formation during epileptogenesis. Decreased PMCA expression levels have also been observed after status epilepticus [86]. The net effect on Ca^2+^ homeostasis in these regions during epileptogenesis, however, was not assessed in these studies.

## 5. Epilepsy and Reactive Oxygen Species

ROS contribute to neuronal damage in a wide range of neurological diseases [87,88,89], including seizures and epilepsy [90,91]. ROS include oxygen radicals such as superoxide, hydroxyl radicals, and hydrogen peroxide (H_2_O_2_) molecules that are by-products of many biological reactions [92]. ROS in excess cause cellular damage due to oxidation induced protein dysfunction and oxidation of DNA and lipids. In an attempt to repair the cell’s DNA, repair enzymes such as the poly(ADP-ribose) polymerase (PARP) excessively consumes ATP and thus stimulates cell death cascades through ATP depletion [93]. It is interesting in this context that PARP activation has recently been shown to contribute to status epilepticus induced mitochondrial function [94]. Whether ROS or sources of ROS are active in the upstream mechanisms of PARP activation has not been determined. ROS are also powerful enhancers of mitochondrial permeability transition pore opening. By stimulation of IP3/ryanodine receptors, sarco/endoplasmic reticulum Ca^2+^-ATPase pump inhibition and inhibition of plasma membrane Ca^2+^ channels, ROS increase intracellular Ca^2+^ levels, which together with ROS, then trigger mitochondrial permeability transition [46] (Figure 2). ROS can also directly interact with membrane lipids triggering lipid peroxidation. This process affects polyunsaturated lipids and increases the instability of the cell membrane [95]. The brain with its high content of polyunsaturated fatty acids is particularly prone to such damage [89].

There is overwhelming evidence supporting a role for ROS in epilepsy [96]. Early studies have investigated brain homogenates [97], whereas with advances in ROS imaging techniques, more sophisticated real time experiments could be performed allowing more detailed cellular analyses [13,98]. Despite this prominent role of ROS in cell homeostasis and in the triggering of mitochondrial dysfunction, previous results of antioxidant therapy in neurologic disease have been mixed. This is most likely due to a lack of characterization of the sources of free radical production and insight into mechanisms of antioxidant protection of the compounds used in these trials [99].

### 5.1. Mitochondria and ROS in Seizures and Epilepsy

Traditionally, mitochondria have been assumed to be the main site of ROS production during seizure activity. Some of this has been initially concluded by the coincidence of mitochondrial membrane depolarization, cellular ROS increases and cellular damage [98]. Complex III has been proposed to be a site of ROS production during seizures, which is based on findings from isolated mitochondria [90]. Using a mitochondria specific ROS probe, we were unable to demonstrate that ROS originate from the mitochondria during seizure like activity at least in the first few minutes of seizure activity [13]. Thus, the exact contribution of mitochondrial ROS to the overall ROS burden during seizures remains to be determined.

A more recent study analyzed hippocampal and parahippocampal tissue samples from 74 patients with drug-refractory temporal lobe epilepsy and found that neuropathological signs of inflammation in patients suffering from hippocampal sclerosis correlated with mitochondrial DNA (mtDNA) mutations [100]. This finding supports the hypothesis that chronic inflammation leads to mitochondrial dysfunction by ROS-mediated mtDNA mutagenesis, which promotes epileptogenesis and neuronal cell loss in patients suffering from mesial temporal lobe epilepsy due to hippocampal sclerosis. This study is interesting since it draws attention to inflammatory ROS. In this model, mitochondria are the targets of ROS induced mutagenesis, with mitochondrial dysfunction occurring as a secondary effect.

### 5.2. NADPH Oxidase Derived ROS and Epilepsy

NADPH oxidase was discovered by studying the respiratory burst in phagocytes and granulocytes [101]. Subsequently, NADPH oxidase expression has been documented in many tissues. There is accumulating evidence highlighting the importance of NADPH oxidase, particularly the isoform NOX2 and NOX4, in brain disease [13,102]. In mammals, seven NADPH isoforms known as NOX1, NOX2, NOX3, NOX4, NOX5, dual oxidase (DUOX1) and DUOX2 have been documented [103,104]. NOX1, NOX2, NOX3 and NOX4 expression has been shown in neurons. NOX4 was demonstrated to play a major role in astrocytes, although NOX2 and NOX1 expression has also been documented [105]. Phagocytes were the cell type that fuelled the discovery of the NADPH oxidase [106]. Thus, besides neurons and astrocytes, microglia, the resident phagocytes of the CNS, unsurprisingly represent a prominent cell type that shows NADPH oxidase activity upon activation [107]. Oligodendrocytes are the only neural cells that do not express NADPH oxidase [104]. Amongst non-neural cells in brain tissue, endothelial cells or pericytes show high expression of NOX4 [108]. In fact, endothelial cells have been shown to be the main site of NOX4 expression [109].

NOX2 has been highlighted to play a role in seizures and epilepsy. This is not surprising since NMDA receptor activation, which has a leading role in epilepsy, has been found to trigger NOX2 assembly and activity [110,111]. We and others have found significant activation of NOX2 during seizure activity and suppression of this enzyme was effective in reducing seizure induced cell death in various epilepsy models [13,112,113,114]. Moreover, NOX2 seems to be involved in status epilepticus induced hypotension since NOX2 was found to be upregulated in the rostral ventrolateral medulla, a key nucleus of the baroreflex loop, which mediated status epilepticus-induced hypotension [115]. In addition, NADPH oxidase was also found to be involved in the vasogenic edema formation during status epilepticus [116]. Analyses of human tissue showed that NOX2 was upregulated in surgical hippocampal specimens from a patient suffering from pharmacoresistant seizures, highlighting that NADPH oxidase plays a role in acquired epilepsy [117]. Table 1 summarizes studies that have looked at the role of NADPH oxidase in epilepsy. It should be noted that some of the studies on the involvement of NADPH oxidase in epilepsy used non-subtype specific NADPH oxidase inhibitors such as apocynin and AEBSF (4-(2-aminoethyl)benzenesulfonyl fluoride hydrochloride) and thus, no conclusion on the isoforms of NADPH oxidase activation can be drawn [14,118]. In addition, there is a lack of studies on knock out animals, which would definitely add to the current evidence.

With regards to the other NADPH oxidase isoforms, besides NOX2, NOX4 has been highlighted as a major source of ROS in acute brain diseases such as stroke [102]. NOX4 is mainly located within intracellular organelles and produces superoxide that is rapidly dismutated into H_2_O_2_ [103]. We have previously shown that NOX2 produces ROS during seizure activity [13], yet NOX2 is particularly active in the early stages of seizure activity. It remains unclear whether other sources of ROS production are involved particularly in later stages of seizure activity, i.e., beyond the first few minutes. We have previously shown that energy depletion and impaired mitochondrial function occurs particularly in prolonged seizure activity [12]. In this context, NOX4-derived ROS are particularly interesting because of the interaction of NOX4 with mitochondrial function and its expression within mitochondrial membranes [119,120].

### 5.3. Other Sources of ROS in Epilepsy

Other sources of ROS have been described in seizures and epilepsy, including xanthine oxidase, cyclooxygenase and lipoxygenase [13,96,121,122]. Xanthine oxidase, an enzyme involved in the catabolism of purines, may be an important source of ROS production during prolonged seizures since breakdown of ATP enhances xanthine oxidase activity. The exact contribution of these sources to ROS production during seizures and epilepsy remains unknown, but it is likely that they are not the main ROS producers in these conditions.

One strategy to decrease the ROS burden during seizure activity is to reduce ROS production by blocking key enzymes; another strategy is to boost ROS scavengers. With regards to the latter, the Nrf2 pathway represents an ideal target.

## 6. Key Regulators of Energy Metabolism and ROS—A Focus on Nrf2 in Seizures and Epilepsy

Nrf2 is a transcription factor that has been shown to regulate both antioxidant defense and intermediary metabolism and thus combines some of the mechanisms outlined above [123,124,125,126]. One of its main negative cytoplasmic regulators is Kelch-like ECH associated protein 1 (KEAP1). KEAP1 is responsible for ubiquitination and proteosomal degradation of Nrf2 and thus tightly controls its activity [127,128,129]. Nrf2 is a potential drug target and there are powerful small molecule inducers that activate Nrf2 by chemically modifying cysteine sensors of KEAP1 or disrupt KEAP1 binding [130,131,132,133]. Some of these small molecule Nrf2 inducers, such as RTA408, are currently being investigated in clinical trials for the treatment of mitochondrial myopathy and Friedreich’s ataxia (Avaliable online: https://clinicaltrials.gov). Moreover, dimethyl fumarate, a drug licensed for use in multiple sclerosis, is an Nrf2 inducer [134], and thus this drug would lend itself to repurposing. Binding of these small molecules to KEAP1 leads to Nrf2 stabilization and translocation to the nucleus where it binds as a heterodimer with a small Maf transcription factor to the antioxidant response element, a specific DNA sequence in the promoter of Nrf2 target genes. This then stimulates transcription of antioxidant proteins such as glutathione-*S*-transferases (GSTs), NAD(P)H:quinone oxidoreductase 1 (NQO1) as well as enzymes involved in glutathione biosynthesis and regeneration [135,136]. Interestingly Nrf2 also controls mitochondrial function by enhancing substrate availability [124]. In addition, it has been reported that treatment of rats with the pharmacological Nrf2 activator sulforaphane promotes resistance of liver mitochondria to redox-regulated MPTP opening [137]. More recently, we have also highlighted an important interaction between NADPH oxidase expression and the Nrf2 pathway where different expression patterns of NOX4 were seen in neuronal cultures with constitutively active Nrf2 in comparison to Nrf2-knockout cells and controls [138]. All these mechanisms of action render Nrf2 an attractive target to treat seizures.

Nrf2 has been highlighted as a target in the treatment of seizures and epilepsy [96,139]. One of the first descriptions of a protective effect of Nrf2 on seizures and epilepsy came from Mazzuferi and colleagues [140]. They screened biosets from epilepsy-related studies and identified Nrf2 as an important transcription factor in epilepsy. They then showed that Nrf2 mRNA expression is increased in human epileptic tissue and in murine brain tissue after status epilepticus. Adeno-associated virus mediated-overexpression of Nrf2 reduced the frequency and duration of seizures induced by pilocarpine in mice. Several studies with small molecule enhancers, i.e., Nrf2 inducers, have been performed. Daily injections of sulforaphane for five days elevated the seizure thresholds to 6 Hz stimulation and fluorothyl-induced seizures. In addition, it protected mice against pilocarpine-induced status epilepticus, demonstrating its efficacy in various epilepsy models [141]. Sulforaphane also suppressed the progression of amygdala kindling, and also ameliorated the cognitive impairment induced by epileptic seizure [142]. An interesting approach was chosen in a recent study by Pauletti and colleagues [143] where they combined the Nrf2 inducer sulforaphane with *N*-acetylcysteine treatment with the rationale that these mechanisms are complementary in increasing glutathione levels, as glutathione is one of the main intracellular antioxidants and thus one of the most potent ROS scavengers within the brain. Sulforaphane at high doses (>100 mg/kg) was shown to lead to sedation, hypothermia, impairment of motor coordination, decrease in skeletal muscle strength, and deaths in addition to offsite effects such as leucopenia [144]. It should be noted, however, that such high concentrations are not necessary for Nrf2 activation and were not administered in the studies that showed a protective effect of sulforaphane. These studies largely used a dose of 5 mg/kg. In addition, efforts are underway to develop highly potent blood–brain barrier permeable Nrf2 inducers and some of the currently available inducers as omaveloxolone (RTA408) have been developed in an attempt to increase blood–brain barrier permeability [145].

**Figure 3:**
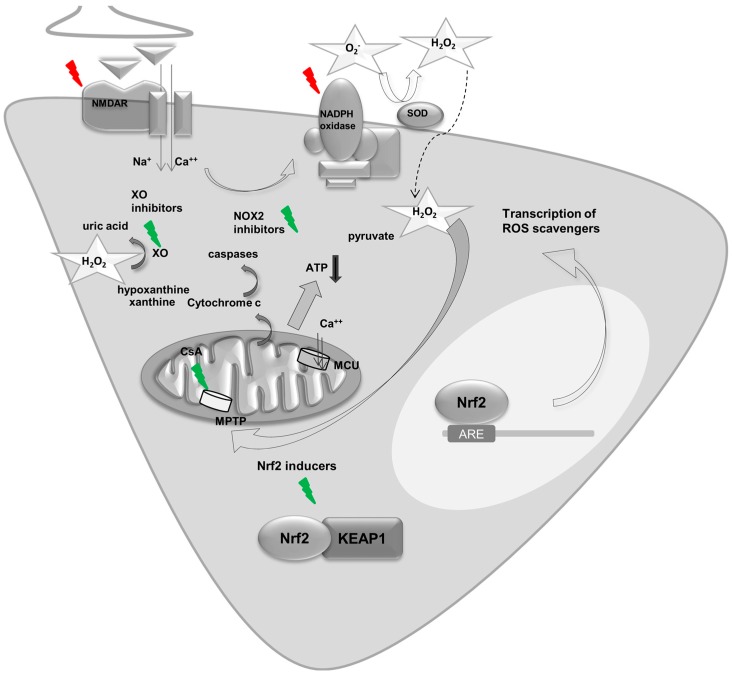
Metabolic and homeostatic changes during seizures and epilepsy. Metabolic and homeostatic changes during seizures and epilepsy and pathways that can be targeted to ameliorate these [96]; NMDA-R: NMDA-Receptor; SOD: superoxide dismutase; ARE: Antioxidant Response element; MCU: mitochondrial Ca^2+^ uniporter; MPTP: mitochondrial permeability transition pore, O_2_^-^: superoxide. KEAP1: Kelch-like ECH-associated protein 1; XO: xanthine oxidase. The red flashes indicate processes which lead to cell death during seizure activity whereas the green flashes represent interventions and targets which reduce cell death during seizure activity.

Figure 3 summarizes metabolic and homeostatic changes during seizures and epilepsy and the pathways that can be targeted to ameliorate these changes.

## 7. Conclusions and Unmet Research Needs

We have highlighted some exciting activity in the field of metabolic and homeostatic changes during seizure activity and in epilepsy. These studies show that mitochondria are both the source and target of metabolic and homeostatic dysfunction during seizures and epilepsy. Ca^2+^ excess is another key candidate for these changes, but the involvement of intracellular calcium stores and particularly the ER in Ca^2+^ excess during seizures and epilepsy remains understudied. Moreover, mitochondrial and ER-Ca^2+^ stores are intimately linked with each other. How this interconnection is in seizures and epilepsy remains an open question. It is known that Ca^2+^ together with ROS induce cell death during seizure activity. We have outlined some ideas about the sources of ROS involved in this process. However, the precise sources of ROS involved remain a matter of debate. It is likely that different sources are active at different time points during seizures and epilepsy, such as has been shown in other diseases, e.g., stroke [146]. With regards to this, we think that investigations into the role of the NADPH oxidase with a focus on the different subtypes is pressing, and such investigations are ideally performed in knock out animals, since current evidence on the role of NADPH oxidase in seizures and epilepsy relies on pharmacological manipulation. This is difficult since NADPH oxidase inhibitors are rarely isoform selective and often not even NADPH oxidase specific [147]. As outlined above, NADPH oxidase isoform expression varies between different brain resident cells, thus another pertinent question is to what extent different cell types contribute to ROS formation during seizure activity. Finally, the Nrf2 pathway has been highlighted as an important pathway that is at the interface of redox and intermediary metabolism within the cell. Nrf2 activation boosts ROS scavengers, but was more recently found to have an effect on ROS producing enzymes such as the NADPH oxidase. Further characterization of this interaction will help to design ideal drug targets and allow for combinations of approaches such as have been recently advocated [143] to combat seizure induced ROS. These are one of the main events leading to cell death and continuous seizures during seizure activity and thus contribute to epilepsy and epilepsy comorbidities.

## Figures and Tables

**Table 1:** Studies on the role of nicotinamide adenine dinucleotide phosphate (NADPH) oxidase in seizures and epilepsy.

Study	Species	Epilepsy Model (In Vivo/Ex Vivo/In Vitro)	NADPH Oxidase Subtype Studied	NADPH Oxidase Inhibition	Main Findings
Zhu et al., 2016 [14]	Mouse	Pentylenetetrazol (PTZ) model (in vivo)	No	Pharmacological (Apocynin)	- Kindling induces NADPH dependent ROS production accompanied by mitochondrial ultrastructural damage - Pharmacological inhibition of NADPH oxidase by apocynin suppressed hippocampal autophagy in the PTZ model
Williams et al., 2015, [118]	Rat	Perforant path stimulation (PPS) model (ex vivo and in vivo)	No	Pharmacological (AEBSF)	- ROS are upregulated and glutathione levels are downregulated in chronic epilepsy - ROS induced cell death in epilepsy can be blocked with NADPH oxidase inhibition with AEBSF
Pecorelli et al., 2015, [117]	Human	Tissue from Patients with drug resistant epilepsy (ex vivo)	Yes (NOX2)	N/A	- p47(phox) and p67(phox) (NOX2) expression in epileptic hippocampus
Kovac et al., 2015 [13]	Rat	Low magnesium model (in vitro)	Yes (NOX2)	Pharmacological (AEBSF, gp-91-tat)	- ROS were generated primarily by NADPH oxidase and later Xanthine oxidase - Inhibition of NADPH or xanthine oxidase reduced seizure-like activity-induced neuronal apoptosis
Kim et al., 2013, [116]	Rat	Pilocarpine induced SE (in vivo)	Yes (NOX2)	Pharmacological (Apocynin)	- Vasogenic edema in SE is mediated via tumor necrosis factor-α (TNF-α) stimulated endothelin-1 (ET-1) release and subsequent endothelial nitric oxide synthase and NADPH oxidase activation - Inhibition of NADPH oxidase attenuated SE induced vasogenic edema
Kim et al., 2013, [112]	Rat	Pilocarpine induced epilepsy (ex vivo and in vivo)	Yes (NOX2)	Pharmacological (Apocynin)	- Pilocarpine-induced seizure increased NOX2 expression in the plasma membrane of hippocampal neurons at 12 h post-insult - Apocynin treatment prevented this increase
Tsai et al., 2012, [115]	Rat	SE due to focal temporal injection of kainic acid (TLSE; ex vivo and in vivo)	Yes (NOX2)	Pharmacological (Apocynin)	- p47^phox^ (NOX2) is upregulated in the rostral ventrolateral medulla, a key nucleus of the baroreflex loop, which mediated SE induced hypotension - Pretreatment with apocynin by microinjection reduced baroreflex-mediated sympathetic vasomotor tone in an experimental model of temporal lobe status epilepticus
Di Maio et al., 2011, [114]	Rat	Pilocarpine induced seizures (in vitro and ex vivo)	Yes (NOX2)	Pharmacological (Apocynin, 6-amino-nicotidamid)	- Apocynin and 6-aminonicotidamid were able to prevent thiol oxidation in vitro - p47^phox^ (NOX2) redistribution to the neuronal cell membrane was seen after pilocarpine treatment (ex vivo)
Pestana et al., 2010, [91]	Rat	Pilocarpine induced SE (in vivo)	No	Pharmacological (Apocynin)	- Apocynin inhibited ROS production and cell death in CA1 and CA3 areas
Patel et al., 2005, [113]	Rat	Kainate model of epilepsy (ex vivo)	Yes (NOX2)	N/A	- Kainate-induced seizures result in the translocation of gp91^phox^ (NOX2) and increased NADPH-driven superoxide production in hippocampal membranes

ROS: reactive oxygen species; NOX2: NADPH Oxidase Subtype 2; CA1 and CA3: hippocampal regions: cornu ammonis 1 and cornu ammonis 3.
